# Deletion of L-Selectin Increases Atherosclerosis Development in ApoE^−/−^ Mice

**DOI:** 10.1371/journal.pone.0021675

**Published:** 2011-07-08

**Authors:** Izabela Rozenberg, Susanna H. M. Sluka, Pavani Mocharla, Anders Hallenberg, Pierre Rotzius, Jan Borén, Nicolle Kränkel, Ulf Landmesser, Lubor Borsig, Thomas F. Lüscher, Einar E. Eriksson, Felix C. Tanner

**Affiliations:** 1 Cardiovascular Research, Institute of Physiology, University of Zurich, Zurich, Switzerland; 2 Center for Integrative Human Physiology, University of Zurich, Zurich, Switzerland; 3 Department of Physiology and Pharmacology, Karolinska Institute, Stockholm, Sweden; 4 Wallenberg Laboratory, Sahlgrenska Academy at Göteborg University, Goteborg, Sweden; 5 Cardiology, Cardiovascular Center, University Hospital Zurich, Zurich, Switzerland; 6 Institute of Physiology, University of Zürich, Zürich, Switzerland; 7 Department of Molecular Medicine and Surgery, Karolinska Institute, Center for Molecular Medicine, Karolinska Hospital, Stockholm, Sweden; Leiden University Medical Center, Netherlands

## Abstract

Atherosclerosis is an inflammatory disease characterized by accumulation of leukocytes in the arterial intima. Members of the selectin family of adhesion molecules are important mediators of leukocyte extravasation. However, it is unclear whether L-selectin (L-sel) is involved in the pathogenesis of atherosclerosis. In the present study, mice deficient in L-selectin (*L-sel* ^−*/*−^) animals were crossed with mice lacking Apolipoprotein E (*ApoE* ^−*/*−^). The development of atherosclerosis was analyzed in double-knockout *ApoE/L-sel* (*ApoE* ^−*/*−^ *L-sel* ^−*/*−^) mice and the corresponding *ApoE* ^−*/*−^ controls fed either a normal or a high cholesterol diet (HCD). After 6 weeks of HCD, aortic lesions were increased two-fold in *ApoE* ^−*/*−^ *L-sel* ^−*/*−^ mice as compared to *ApoE* ^−*/*−^ controls (2.46%±0.54% vs 1.28%±0.24% of total aortic area; p<0.05). Formation of atherosclerotic lesions was also enhanced in 6-month-old *ApoE* ^−*/*−^ *L-sel* ^−*/*−^ animals fed a normal diet (10.45%±2.58% vs 1.87%±0.37%; p<0.05). In contrast, after 12 weeks of HCD, there was no difference in atheroma formation between *ApoE* ^−*/*−^ *L-sel* ^−*/*−^ and *ApoE* ^−*/*−^ mice. Serum cholesterol levels remained unchanged by L-sel deletion. Atherosclerotic plaques did not exhibit any differences in cellular composition assessed by immunohistochemistry for CD68, CD3, CD4, and CD8 in *ApoE* ^−*/*−^ *L-sel* ^−*/*−^ as compared to *ApoE* ^−*/*−^ mice. Leukocyte rolling on lesions in the aorta was similar in *ApoE* ^−*/*−^ *L-sel* ^−*/*−^ and *ApoE* ^−*/*−^ animals. *ApoE* ^−*/*−^ *L-sel* ^−*/*−^ mice exhibited reduced size and cellularity of peripheral lymph nodes, increased size of spleen, and increased number of peripheral lymphocytes as compared to *ApoE* ^−*/*−^ controls. These data indicate that L-sel does not promote atherosclerotic lesion formation and suggest that it rather protects from early atherosclerosis.

## Introduction

Endothelial activation and subsequent accumulation of leukocytes is a key event in early atherosclerosis [1]. The selectin family of adhesion molecules mediates initial rolling and tethering of inflammatory cells at sites of activated endothelium [2], [3], [4], [5], [6]. The family consists of the three closely homologous glycoproteins E-selectin (E-sel), P-selectin (P-sel), and L-selectin (L-sel), that all bind glycoproteins and glycolipids bearing sialyl Lewis X (sLeX) in a calcium-dependent manner [7], [8]. Upon stimulation, E-sel is expressed on endothelial cells, while P-sel is expressed in both endothelial cells and platelets. L-sel, on the other hand, is constitutively expressed on the majority of leukocytes [6].

L-sel exhibits adhesive as well as signaling functions [9], [10] and is particularly important for lymphocyte homing to secondary lymphoid organs [5], [11]. Indeed, animals lacking L-sel display an altered size of secondary lymphoid tissues and increased numbers of peripheral lymphocytes [11], [12], [13]. Moreover, L-sel deficient mice show reduced leukocyte rolling along cytokine-stimulated endothelium *in vivo*. This is well documented in venules in the microcirculation and primarily depends on a lack of L-sel-mediated interactions between leukocytes regulating capture of cells from the free flow [14], [15]. Indeed, whether functional L-sel ligand activity is regularly upregulated on inflamed endothelium is still under debate [16].

Since the selectins are known to regulate leukocyte recruitment in inflammation, they are interesting candidates to study in the context of atherogenesis. Indeed, mice deficient in E- and P-sel display attenuated development of atherosclerosis [2], [17]. Moreover, lymphocyte recruitment to the aortic wall during atherosclerosis development is partially L-sel dependent [18]. However, there are no *in vivo* reports addressing the impact of L-sel for the development of atherosclerotic lesions *in vivo*. In this study, L-selectin deficient (*L-sel* ^−*/*−^) mice were crossed with Apolipoprotein E deficient mice (*ApoE* ^−*/*−^) to investigate the relevance of L-sel on both early and advanced stages of atherosclerosis.

## Results

### L-selectin attenuates early, but not advanced atherosclerosis

**Figure 1:**
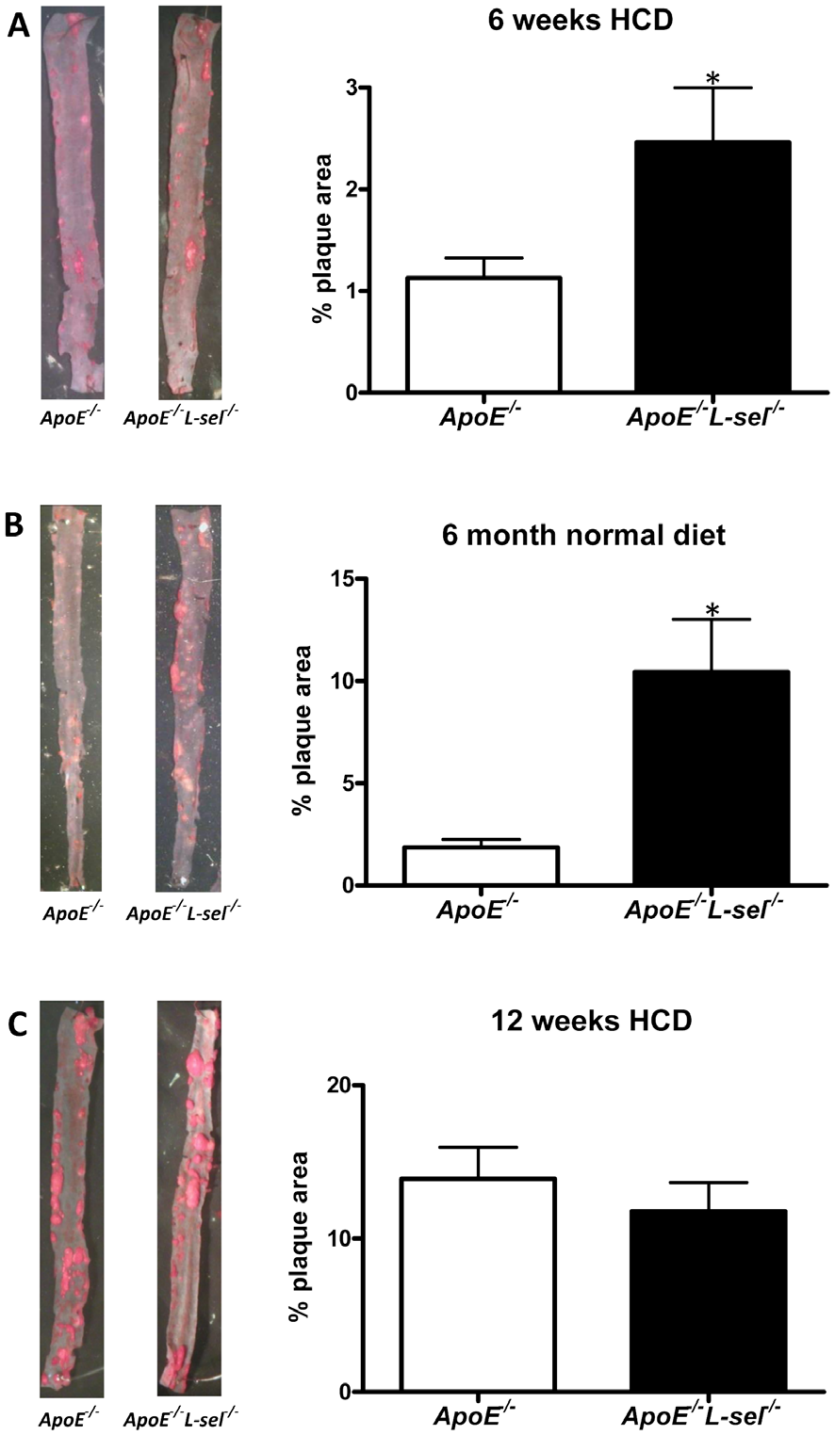
L-sel modulates atherosclerosis development. Representative images of aortas stained with Oil-Red-O (ORO). Atherosclerotic lesion area is expressed as percentage of total aortic area. Compared to *ApoE* ^−*/*−^ controls, there is an increased atherosclerotic burden in **A**) 12 week old *ApoE* ^−*/*−^ *L-sel* ^−*/*−^ animals after 6 weeks of HCD (n = 11–15; *p<0.05); and **B**) 6 month old *ApoE* ^−*/*−^ *L-sel* ^−*/*−^ animals after 6 months of a normal diet (n = 5–9; *p<0.05), but not **C**) 18 week old *ApoE* ^−*/*−^ *L-sel* ^−*/*−^ animals after 12 weeks of HCD (n = 10–15; p = n.s.).

The development of atherosclerosis was monitored in descending aortas of mice with or without L-sel. In 12 week old *ApoE* ^−*/*−^ *L-sel* ^−*/*−^ animals fed a HCD for 6 weeks, the percentage of the aorta occupied by atherosclerotic plaques was two fold higher than in age- and diet-matched *ApoE* ^−*/*−^ controls (2.46%±0.54% vs 1.13%±0.19%, respectively; p<0.05; Fig. 1A). The effect of L-sel deletion was even more pronounced in 6 month old animals fed a normal diet. Under these conditions, *ApoE* ^−*/*−^ *L-sel* ^−*/*−^ mice had 10.45±2.58% of the descending aorta covered by plaques as compared to 1.87±0.37% in *ApoE* ^−*/*−^ controls (p<0.05; Fig. 1B). In contrast, the atherosclerotic burden in 18 week old *ApoE* ^−*/*−^ *L-sel* ^−*/*−^ animals fed a HCD for 12 weeks (11.80±1.86%) was similar to that of *ApoE* ^−*/*−^ controls (13.89±2.06%; p = n.s.; Fig. 1C). There was no difference in plasma cholesterol levels between double knockout and control mice in any of the groups (p = n.s.; Table S1). Expression of E-sel did not differ in *ApoE* ^−*/*−^ controls and *ApoE* ^−*/*−^ *L-sel* ^−*/*−^ animals during atherosclerotic lesion formation (p = n.s.; Fig. S1A). P-sel expression was significantly increased in arteries of *ApoE* ^−*/*−^ mice after 6 weeks of HCD compared to *ApoE* ^−*/*−^ *L-Sel* ^−*/*−^ mice. (p<0.05; Fig. S1B) Vascular smooth muscle cell accumulation was similar in the two groups (p = n.s.; Fig. S1D). Staining for collagen exhibited a minor increase in plaques from *ApoE* ^−*/*−^ *L-sel* ^−*/*−^ animals as compared to *ApoE* ^−*/*−^ controls (p<0.05; Fig. S1C).

### L-selectin does not influence leukocyte capture and rolling in atherosclerosis

**Figure 2:**
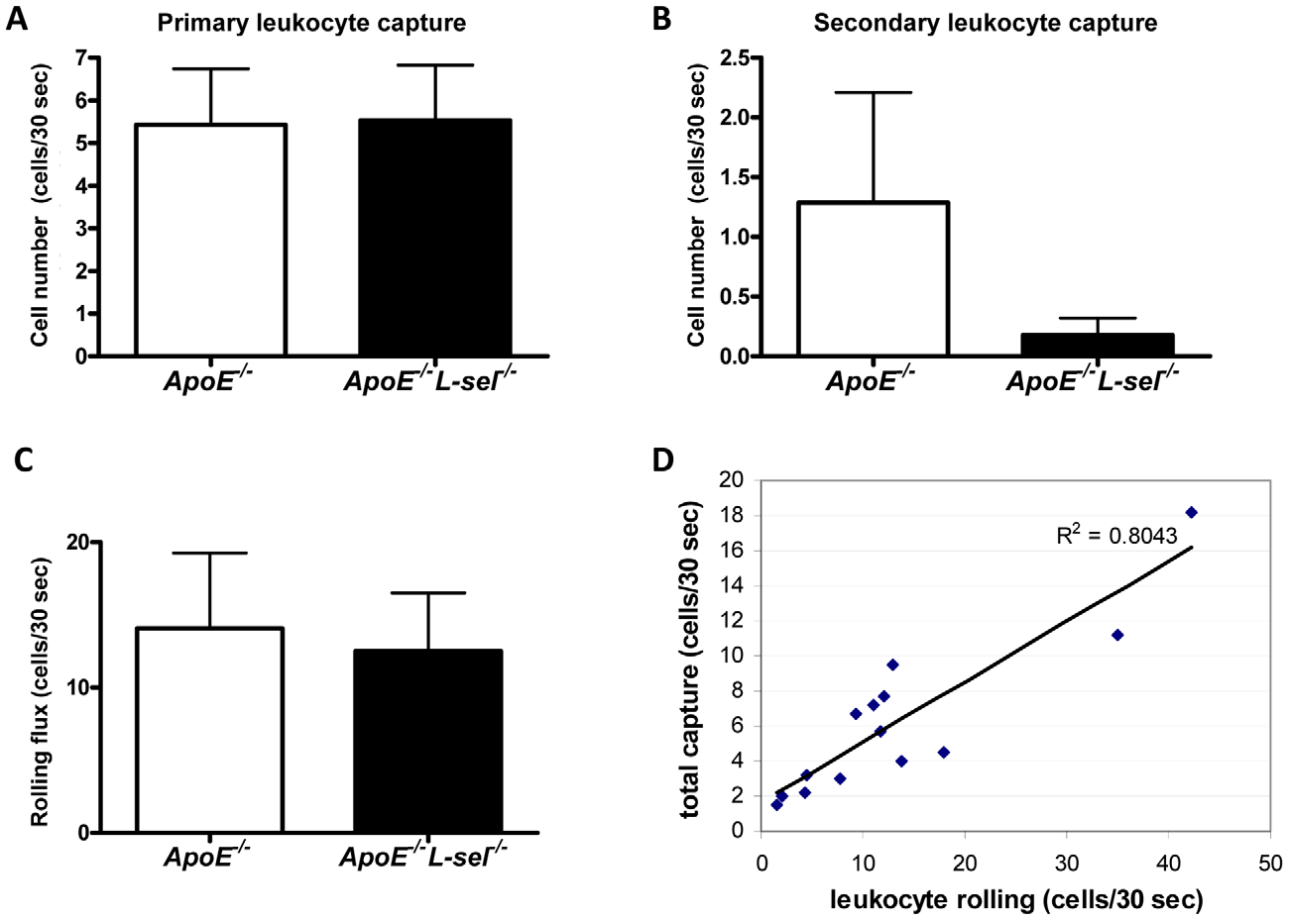
L-sel does not affect leukocyte capture and rolling in atherosclerosis. There was no difference between *ApoE* ^−*/*−^ and *ApoE* ^−*/*−^ *L-sel* ^−*/*−^ animals in either **A**) primary or **B**) secondary leukocyte capture (n = 7; p = n.s.). **C**) Leukocyte rolling in atherosclerotic aorta was not affected by the presence or absence of L-sel (n = 7; p = ns). **D**) Total leukocyte capture was proportional to leukocyte rolling flux (R = 0.8).

Leukocyte capture and rolling were assessed using intravital microscopy. There was no difference between *ApoE* ^−*/*−^ and *ApoE* ^−*/*−^ *L-sel* ^−*/*−^ animals in primary leukocyte capture directly to the endothelium from the free flow (5.4±1.3 cells vs 5.5±1.3 cells, respectively; p = n.s.; Fig. 2A). Secondary capture mediated by interactions between leukocytes was low in *ApoE* ^−*/*−^ as well as in *ApoE* ^−*/*−^ *L-sel* ^−*/*−^ mice (1.28±0.92 cells vs 0.17±0.14 cells, respectively; p = n.s.; Fig. 2B). Correspondingly, there was no difference in the total number of cells rolling along the aortic endothelium in *ApoE* ^−*/*−^ controls and *ApoE* ^−*/*−^ *L-sel* ^−*/*−^ mice (p = n.s.; Fig. 2C). Total capture correlated with the number of rolling cells (Fig. 2D).

### L-selectin does not influence leukocyte accumulation in atherosclerotic plaques

**Figure 3:**
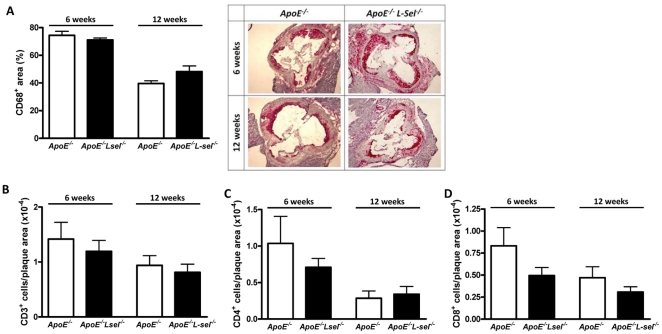
L-sel does not affect leucocyte accumulation in atherosclerotic plaques. Immunohistochemical analysis of **A**) macrophages (CD68^+^), **B**) T-lymphocytes (CD3^+^), **C**) T helper cells (CD4^+^), and **D**) T-cytotoxic cells (CD8^+^) in aortic arch after 6 and 12 weeks of HCD (n = 5–13; p = n.s.).

Immunohistochemistry on the aortic root was performed to monitor the composition of atherosclerotic plaques following L-sel deletion. Macrophages were visualized with anti-CD68 antibody, lymphocytes with anti-CD3 antibody, T-helper and T-cytotoxic cells with anti-CD4 and anti-CD8 antibodies, respectively. L-sel deletion did not result in an altered CD68 positive area in either early (71.10%±1.36% vs 74.44%±2.90%; p = n.s.; Fig. 3A) or advanced atherosclerosis (48.20%±4.13% vs 39.62%±1.99%; p = n.s.; Fig. 3A). Similarly, there was no difference between *ApoE* ^−*/*−^ and *ApoE* ^−*/*−^ *L-sel* ^−*/*−^ mice in the number of CD3 (p = n.s.; Fig. 3B), CD4 (p = n.s.; Fig. 3C), and CD8 positive cells (p = n.s.; Fig. 3D) after 6 and 12 weeks of HCD. Increased duration of HCD resulted in a decreased plaque area occupied by macrophages in both *ApoE* ^−*/*−^ and *ApoE* ^−*/*−^ *L-sel* ^−*/*−^ mice (p<0.05; Fig. 3A). In contrast, no significant difference in T-cell accumulation was observed after 6 and 12 weeks of HCD (p = n.s.). Cytokine expression was similar in aortas of *ApoE* ^−*/*−^ *L-sel* ^−*/*−^ mice as compared to *ApoE* ^−*/*−^ controls after 6 and 12 weeks of HCD (p = ns; Table 1). In contrast, the animals exhibited enhanced cytokine expression after 12 as compared to 6 weeks of HCD irrespective of the genotype (p<0.05; Table 1). The majority of circulating cytokines exhibited similar plasma levels in *ApoE* ^−*/*−^ and *ApoE* ^−*/*−^ *L-sel* ^−*/*−^ mice after 6 weeks of HCD. Interestingly, the level of the chemotactic cytokine MCP-1 was four fold elevated in plasma of *ApoE* ^−*/*−^ *L-sel* ^−*/*−^ mice (p<0.05; Table 2).

**Table 1:** mRNA expression (normalized to S12 expression) of different cytokines in atherosclerotic plaques is not affected by L-sel after 6 and 12 weeks of HCD.

	6 weeks		12 weeks	
**expression level**	*ApoE* ^−^ */* ^−^	*ApoE* ^−^ */* ^−^ *L-sel* ^−^ */* ^−^	*ApoE* ^−^ */* ^−^	*ApoE* ^−^ */* ^−^ *L-sel* ^−^ */* ^−^
**MCP-1 (×10^−2^)**	2.02±0.58	2.59±0.37	5.22±1.47	5.95±.20
**TNFα (×10^−3^)**	1.40±0.39	1.40±0.15	3.90±1.30	4.2±1.01
**INFγ (×10^−4^)**	4.51±1.8	3.88±0.7	18.71±8.00	25.45±10.30
**Mip-1 (×10^−2^)**	3.44±0.59	2.84±0.36	5.88±1.88	3.14±0.65
**IL-4 (×10^−4^)**	4.43±1.20	3.51±0.40	11.01±3.70	10.11±2.70
**IL-6 (×10^−3^)**	1.11±0.22	1.34±0.25	2.32±0.64	2.22±0.59
**IL-10 (×10^−4^)**	2.22±1.00	2.79±0.40	6.51±1.60	4.79±1.30

**Table 2:** Plasma MCP-1 is elevated in *ApoE* ^−*/*−^ *L-sel* ^−*/*−^ mice compared to *ApoE* ^−*/*−^ mice after 6 weeks of HCD, while the other plasma cytokines are not affected.

plasma concentration (pg/ml) ± st.dev.	*ApoE^−^/^−^*	*ApoE^−^/^−^ L-Sel^−^/^−^*	p
**GM-CSF**	10.8±3.3	11.6 ±6.8	0.805
**IFN-γ**	63.5±35.7	43.2±16.0	0.274
**IL-1β**	20.9±11.1	15.1±4.2	0.307
**IL-2**	13.2±4.3	12.0±5.5	0.694
**IL-4**	19.3±6.2	16.4±2.1	0.349
**IL-5**	192.7±71.0	125.6±38.8	0.093
**IL-6**	37.9±18.0	61.6±20.2	0.070
**IL-10**	83.5±11.6	80.1±13.7	0.664
**IL-12**	47.2±8.9	46.3±4.7	0.844
**IL-17**	132.0±49.1	89.2±10.1	0.090
**MCP-1**	86.5±79.1	332.7±230.5	0.035
**RANTES**	16.9±11.1	8.9±5.9	0.183
**VEGF**	43.2±13.5	65.0±30.0	0.141

### Increased number of circulating lymphocytes in *L-sel* ^−*/*−^ mice

**Figure 4:**
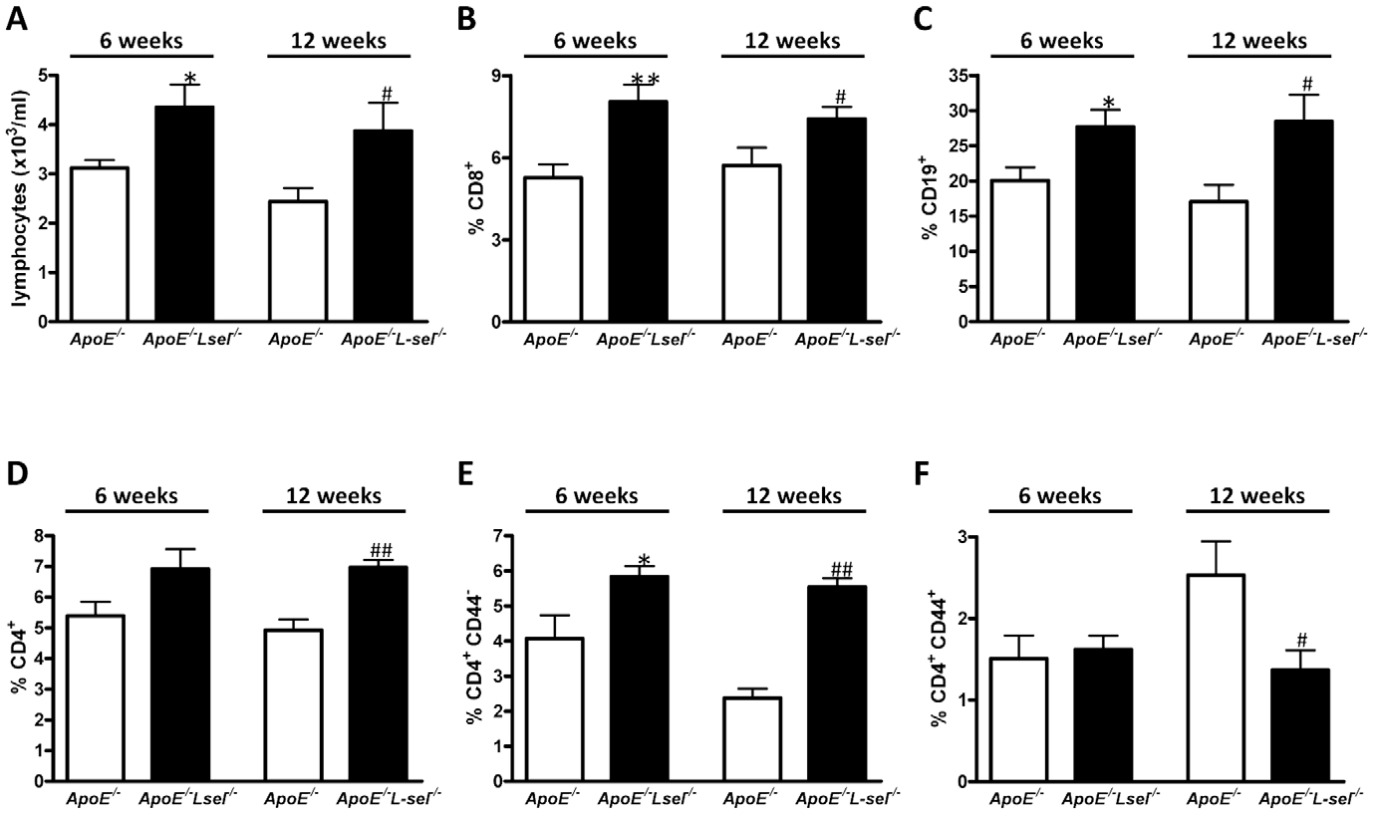
L-sel decreases the number of circulating lymphocytes. *ApoE* ^−*/*−^ *L-sel* ^−*/*−^ animals exhibit increased number of **A**) total blood lymphocytes (n = 5–8; ^*^p<0.05), **B**) T-cytotoxic cells (CD8^+^; n = 6–7; ^**^p<0.01, ^#^p<0.05), **C**) B lymphocytes (CD19^+^; n = 5–7; ^*^p<0.05, ^#^p<0.05), **D**) total T-helper cells (CD4^+^; n = 6–7; ^#^p<0.0), **E**) naive T-helper cells (CD4^+^CD44^−^; n = 5–7; ^*^p<0.05; ^##^p<0.0001) after 6 and 12 weeks of HCD, respectively. **F**) The number of activated T-cells (CD4^+^CD44^+^) remained unchanged after 6 weeks of HCD (n = 5–7; p = n.s.) and decreased after 12 weeks of HCD (n = 5–7; ^*^p<0.05) as compared to control.

*ApoE* ^−*/*−^ *L-sel* ^−*/*−^ animals exhibited a 1.4 fold and 1.6 fold increased number of blood lymphocytes as compared to *ApoE* ^−*/*−^ controls after 6 and 12 weeks of HCD, respectively (p<0.05; Fig. 4A). Consistent with this observation, there was an increased number of CD8^+^ and CD19^+^ cells in *ApoE* ^−*/*−^ *L-sel* ^−*/*−^ mice irrespectively of the duration of HCD (p<0.05; Fig. 4B and C). Moreover, there was tendency towards an increased number of CD4^+^ cells in *ApoE* ^−*/*−^ *L-sel* ^−*/*−^ mice after 6 weeks of HCD (p = 0.24), which was significant after 12 weeks of this diet (p<0.05; Fig. 4D). The increased number of circulating lymphocytes in *ApoE* ^−*/*−^ *L-sel* ^−*/*−^ mice was associated with an increased number of naive T helper cells (CD4^+^CD44^−^; p<0.05; Fig. 4E) after 6 and 12 weeks of HCD. The number of activated T helper cells (CD4^+^CD44^+^) did not differ after 6 weeks, but was lower in *ApoE* ^−*/*−^ *L-sel* ^−*/*−^ as compared to *ApoE* ^−*/*−^ mice after 12 weeks of HCD (p<0.05; Fig. 4F). No significant difference in the circulating leukocyte profile was observed after 6 and 12 weeks of HCD in any of the genotypes.

A significant reduction in size (p<0.05; Fig. S2A) and cellularity (p<0.05; Fig. S2B) of peripheral lymph nodes was observed in *ApoE* ^−*/*−^ *L-sel* ^−*/*−^ as compared to *ApoE* ^−*/*−^ mice after 6 and 12 weeks of HCD. In contrast, the spleen was 30% larger in L-sel deficient mice at both time-points (p<0.05; Fig. S3A). The increased spleen size was not associated with an altered cellularity, cell composition (p = n.s.; Fig. S3B and C) or an altered cytokine expression (p = n.s.; data not shown).

## Materials and Methods

### Mice

The development of atherosclerosis in *ApoE* ^−*/*−^ *L-sel* ^−*/*−^ mice was studied by two independent experiments. In the first set, *L-sel* ^−*/*−^ mice backcrossed to C57Bl/6 background for 9 generations were used. In the other set, *L-sel* ^−*/*−^ mice were purchased from Jackson Lab and backcrossed to C57Bl/6 background for 5 generations. *L-sel* ^−*/*−^ mice were cross-bred with *ApoE* ^−*/*−^ mice to generate *ApoE* ^−*/*−^ *L-sel* ^−*/*−^ and littermate *ApoE* ^−*/*−^ controls. In the first set, 6 week old males were fed a HCD (Clinton-Cybulski diet, 1.25% cholesterol, Research Diets #D12108) for 6 or 12 weeks. In the other set, male *ApoE* ^−*/*−^ *L-sel* ^−*/*−^ animals and controls were fed a normal diet for 6 months before analysis. All animal experiments were approved by the appropriate authorities.

### Quantification of atherosclerosis development

Under anesthesia, blood was collected through right ventricular puncture. Mice were then perfused with ice-cold saline. Aortas were harvested for RNA isolation, en face, and histological analysis. The thoracic and abdominal part of the aortas were fixed overnight with 4% paraformaldehyde (PFA), washed 3 times with ice-cold PBS, and stained for 3 hours with Oil red-O (Sigma #O9755). Quantification of aortic plaque area was performed using AnalysisFIVE software manually by an investigator blinded to the genotypes.

### Plasma triglyceride and cholesterol level

Plasma cholesterol level was determined using Infinity™ Cholesterol (Thermo Electron Corporation Standard #TR13421) and MC Cal (Abbott #1E65-02), plasma triglycerides using Infinity™ Triglycerides (Thermo Electron Corporation Standard #TR22421) and MC Cal (Abbott #1E65-02). The distribution of lipids within the plasma lipoprotein fractions was assessed by fast-performance liquid chromatography (FPLC) gel filtration using a Superose 6 HR 10/30 column (Pharmacia).

### Immunohistochemistry

Aortic roots were harvested, mounted in O.C.T. compound (Tissue-Tek #62550-01) and frozen at −20°C. 8 µm-thick slices were fixed with 4% PFA and stained with anti-mouse CD3, CD4 CD8, or CD68 antibodies (Serotec), followed by incubation with alkaline phosphatase–conjugated secondary antibody (Jackson ImmunoResearch). Aortic arches were fixed with 4% formalin and embedded in paraffin. Sections were stained with anti-mouse α-SMA (clone 1A4, SIGMA), E-selectin (abcam), and P-selectin (LSBio). Percentages of stained area were quantified with AnalySIS-FIVE program.

### Intravital microscopy

18 week old male mice fed with a HCD for 12 weeks were anesthetized with isofluran. The aorta was prepared as described previously [19]. Briefly, the abdomen was opened by a midline incision and the intestines were retracted. The peritoneum was then dissected to expose the abdominal aorta. The exposed tissue was superfused with a thermostated (37°C) bicarbonate-buffered saline solution. Microscopic observations were made using an intravital microscope (Leitz Biomed) with a water immersion objective (Leitz SW 25×). Epi-illumination fluorescence microscopy (Leitz Ploem-o-pac, filter block M2 illuminated by a cooled infrared filtered lamp (Osram HBO 200W/4)) was started 2 minutes after labeling of circulating leukocytes with an intravenous injection of rhodamine 6G (0.3 mg/ml, 0.67 mg/kg). Images were televised and recorded on videotape using a VNC-703 video camera. Leukocyte rolling flux was determined as the average number of leukocytes rolling within a 10000 µm^2^ area during 30 seconds within a total observation time of at least 180 seconds. Leukocyte capture was determined as the number of leukocytes that initiated rolling within a 10000 µm^2^ area during 30 seconds [20]. Leukocyte capture in contact with or 50 µm downstream of rolling or adherent leukocytes were regarded as secondary, all other capture was regarded as primary.

### Facs

Blood cells were stained with fluorescently labeled anti-mouse CD4 (PE-conjugated, clone RM4-5), CD8 (PE-Cy7-conjugated, clone H35-17.2), CD19 (PE-Cy7-conjugated, clone 1D3), or CD44 (APC-conjugated, clone IM7) antibodies (Pharmingen) for 30 minutes at 4°C. Erythrocytes were lyzed following staining using commercially available lysis buffer (BD #555899). Data were collected using DIVAII (BD), and FACS analysis was performed using FlowJo software.

### Statistical analysis

The results were expressed as mean ± S.E.M. Comparison of means was carried out by Student's t-test or ANOVA in case of multiple comparisons. For each experiment, P<0.05 was accepted as statistically significant.

## Discussion

Accumulation of leukocytes in the arterial wall is an important pathogenic event in atherogenesis. It is well documented that the selectin family of adhesion molecules mediates initial attachment of leukocytes to activated endothelium, representing the first step of leukocyte emigration into sites of inflammation [2], [6]. Correspondingly, L-sel may play a role in the migration of leukocytes to atherosclerotic lesions and data have been presented supporting that lymphocyte recruitment during atherosclerosis development is partially L-sel dependent [18]. Thus, we hypothesized that deletion of L-sel might attenuate the development of atherosclerosis due to inhibition of leukocyte rolling and capture. To test this hypothesis, we compared atherogenesis in *ApoE* ^−*/*−^ *L-sel* ^−*/*−^ mice with that of *ApoE* ^−*/*−^ controls [21]. Interestingly, the data show that L-sel does not promote atherosclerotic lesion formation in *ApoE* ^−*/*−^ mice. On the contrary, genetic deficiency in *L-sel* resulted in a significant increase in lesion formation, at least during early stages of the disease. Indeed, after 6 month of normal diet, atherosclerosis was relatively advanced in the absence of L-sel, while plaque burden was still low in the *ApoE* ^−*/*−^ control group and comparable to the *ApoE* ^−*/*−^ control animals after 6 weeks of HCD. Thus, both feeding protocols induce an early stage of the disease; however, the absence of L-sel results in a strong increase of atherosclerosis. In line with this, there was no decrease in leukocyte rolling between *ApoE* ^−*/*−^ *L-sel* ^−*/*−^ and *ApoE* ^−*/*−^ control mice in the atherosclerotic aorta. These observations indicate that other members of the selectin family are sufficient to maintain leukocyte-endothelium interactions under conditions of L-sel deletion [17], [22], [23]. In line with this interpretation, expression of E-sel and P-sel were not upregulated in the absence of L-sel during atherosclerotic lesion formation. Previous data reveal that the effect of combined deficiency of P- and E-sel has an effect on rolling and recruitment in inflammation which is much stronger than that seen in mice deficient in L-sel [22], [23]. Blockage of P-sel also virtually abolishes interactions between leukocytes and endothelium in the atherosclerotic aorta and inhibition of E-sel stabilizes leukocyte rolling under these conditions [2] supporting that E- and P-sel are key mediators of initial leukocyte attachment in arteries. Combined deficiency in E-sel and P-sel also strongly reduces the formation of atherosclerotic lesions [17]. In contrast, as previously indicated [14], L-sel-dependent secondary capture does not increase rolling on atherosclerotic endothelium. Nonetheless, L-sel increases rolling in venules in the microcirculation [11], [14], [24], which has been shown to be dependent mainly on interactions between leukocytes. Ligands for L-sel are only expressed by endothelium in secondary lymphoid tissues and, under certain circumstances, also by chronically inflamed systemic endothelium [18], [25]. Interestingly, data from a previous study suggested that L-sel dependent accumulation of lymphocytes in arteries occurs almost exclusively from the adventitial side of the vessel suggesting that L-sel influences recruitment from the vasa vasorum [18]. This apparent role of L-sel could be mediated by both direct interactions between leukocytes and endothelium as well as secondary capture interactions. Thus, it is possible that L-sel influences rolling and recruitment in other parts of the vascular wall than in the arterial lumen. A strong argument against this interpretation is that plaques from *ApoE* ^−*/*−^ *L-sel* ^−*/*−^ mice exhibited similar numbers of macrophages and T cells as compared to lesions from *ApoE* ^−*/*−^ controls. Ideally, the cellular composition of the plaque should be examined in the descending aorta, i.e. at that site of the aorta in which significant differences in plaque size were noted. As the atherosclerotic alterations in the descending aorta are focal, it is virtually impossible to cut the descending aorta at the same site and find a plaque of similar size to assess and compare its composition. For this reason, cellular plaque composition was studied in the aortic sinuses, i.e. at a site where plaques of similar size could consistently be detected. It has been observed that even if plaque size is similar at the level of the aortic root, differences in plaque composition can be detected [26].

No difference in cytokine expression in atherosclerotic vessel walls from *ApoE* ^−*/*−^ *L-sel* ^−*/*−^ and *ApoE* ^−*/*−^ mice was detected indicating a similar extent of local inflammation without or with L-sel. The enhanced aortic cytokine levels in animals treated with HCD for 12 weeks as compared to those treated for 6 weeks is consistent with a more advanced stage of atherosclerosis in these mice and did not differ between strains. Hence, alterations in local inflammation do not seem to account for the atheroprotective actions of L-sel. In line with these observations, plasma cytokine levels were similar in *ApoE* ^−*/*−^ *L-sel* ^−*/*−^ and *ApoE* ^−*/*−^ mice. In plasma of *ApoE* ^−*/*−^ *L-sel* ^−*/*−^ mice fed a HCD for 6 weeks the levels of the chemotactic cytokine MCP-1 were elevated compared to *ApoE* ^−*/*−^ mice, which most likely reflects the increased plaque burden in these animals.

Atherosclerosis does not only affect the wall of blood vessels, but also provokes changes at the systemic level [27], [28]. Deletion of L-sel resulted in abnormal systemic leukocyte distribution, which could potentially affect atherosclerosis development [29]. Both size and cellularity of peripheral lymph nodes were decreased in *ApoE* ^−*/*−^ *L-sel* ^−*/*−^ mice as compared to *ApoE* ^−*/*−^ controls, which is consistent with the observation that migration of naive lymphocytes into peripheral lymph nodes is impaired in L-sel deficient mice [11], [13]. Likely due to compensation for this impaired migration into tissues, L-sel deficiency results in increased numbers of circulating lymphocytes. Hence, it is possible that the enhanced atherosclerosis in *ApoE* ^−*/*−^ *L-sel* ^−*/*−^ mice is driven by more abundant circulating proatherogenic cells, in particular because migration of these cells into lesions appears not to be impaired by lack of L-sel [2], [30].

In conclusion, this study demonstrates that absence of L-sel does not inhibit atherosclerosis but rather augments the early stages of atherogenesis. This effect is not associated with reduced leukocyte rolling or accumulation nor with altered cytokine production in atherosclerotic plaques.

## Supporting Information

Figure S1 Histochemical and immunohistochemical stainings of aortic arches from mice fed a HCD for 6 weeks: **A**) Similar expression of E-sel in *ApoE* ^−*/*−^ and *ApoE* ^−*/*−^ *L-sel* ^−*/*−^ mice. **B**) Increased expression of P-sel in *ApoE* ^−*/*−^ compared to *ApoE* ^−*/*−^ *L-sel* ^−*/*−^ mice (p<0.05) **C**) Collagen area is increased in *ApoE* ^−*/*−^ *L-Sel* ^−*/*−^ mice compared to *ApoE* ^−*/*−^ mice (p<0.01) **D**) Smooth muscle cell area is similar in *ApoE* ^−*/*−^ and *ApoE* ^−*/*−^ *L-sel* ^−*/*−^ mice.
(TIF)

Figure S2 Decreased **A**) size (n = 5–6; **p<0.01; ^##^p<0.01) and **B**) cellularity (n = 5–6; *p<0.05; ^#^p<0.05) of peripheral lymph nodes (LN) in mice lacking L-sel after 6 and 12 weeks of HCD.
(TIF)

Figure S3 **A**) Spleen size is increased upon L-sel deletion (n = 6; **p<0.01; ^#^p<0.05). **B**) Spleen cellularity is not affected by L-sel deletion (n = 5–6; *p = n.s.) after 6 and 12 weeks of HCD. **C**) Increased size but unchanged cell composition (table; % of leukocytes ± SEM) of spleens from animals after 6 month of normal diet.
(TIF)

Table S1 Plasma cholesterol and triglyceride levels (n = 5–12; p = n.s.) after normal diet or 6 and 12 weeks of HCD.
(DOC)
