# Supplementary figures and images for: Deletion of L-Selectin Increases Atherosclerosis Development in ApoE−/− Mice
Source: PLoS One. 2011 Jul 8;6(7):e21675. doi: 10.1371/journal.pone.0021675 (PMC3132176; doi:10.1371/journal.pone.0021675)

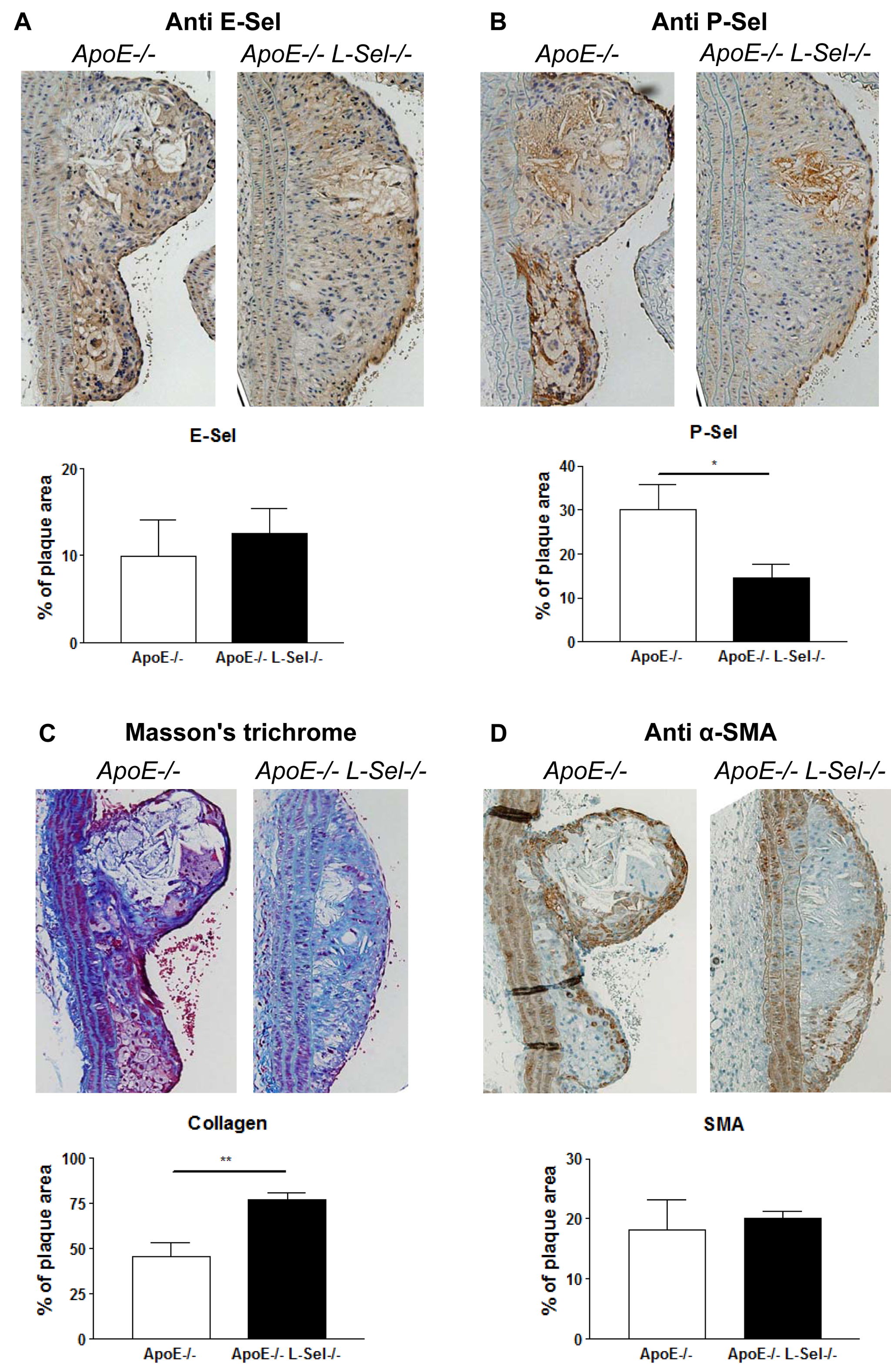

Supplement: Figure S1 — Histochemical and immunohistochemical stainings of aortic arches from mice fed a HCD for 6 weeks: A) Similar expression of E-sel in ApoE −/− and ApoE −/− L-sel −/− mice. B) Increased expression of P-sel in ApoE −/− compared to ApoE −/− L-sel −/− mice (p<0.05) C) Collagen area is increased in ApoE −/− L-Sel −/− mice compared to ApoE −/− mice (p<0.01) D) Smooth muscle cell area is similar in ApoE −/− and ApoE −/− L-sel −/− mice. (TIF) [file pone.0021675.s001.tif]

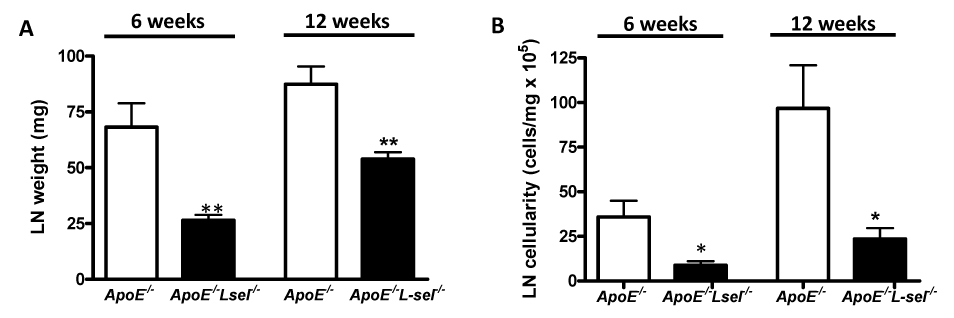

Supplement: Figure S2 — Decreased A) size (n = 5–6; **p<0.01; ##p<0.01) and B) cellularity (n = 5–6; *p<0.05; #p<0.05) of peripheral lymph nodes (LN) in mice lacking L-sel after 6 and 12 weeks of HCD. (TIF) [file pone.0021675.s002.tif]

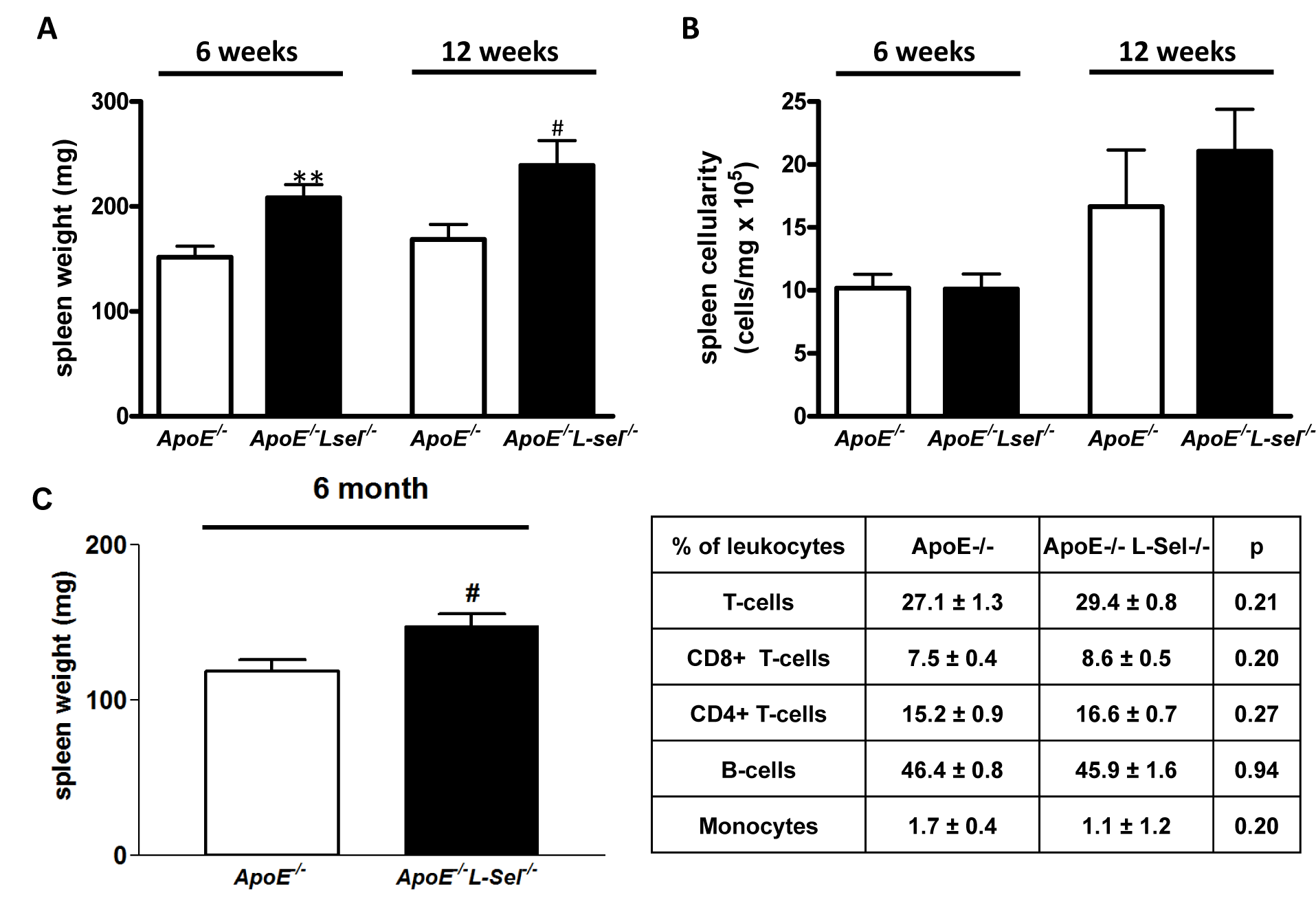

Supplement: Figure S3 — A) Spleen size is increased upon L-sel deletion (n = 6; **p<0.01; #p<0.05). B) Spleen cellularity is not affected by L-sel deletion (n = 5–6; *p = n.s.) after 6 and 12 weeks of HCD. C) Increased size but unchanged cell composition (table; % of leukocytes ± SEM) of spleens from animals after 6 month of normal diet. (TIF) [file pone.0021675.s003.tif]
