# Supplementary material for: Deletion of L-Selectin Increases Atherosclerosis Development in ApoE−/− Mice
Source: PLoS One. 2011 Jul 8;6(7):e21675. doi: 10.1371/journal.pone.0021675 (PMC3132176; doi:10.1371/journal.pone.0021675)
Supplement: Table S1 — Plasma cholesterol and triglyceride levels (n = 5–12; p = n.s.) after normal diet or 6 and 12 weeks of HCD. (DOC) [file pone.0021675.s004.doc]

|  | **no** | | **6 weeks** | | **12 weeks** | |
| --- | --- | --- | --- | --- | --- | --- |
| **mmol/l** | *ApoE-/-* | *ApoE-/-L-sel-/-* | *ApoE-/-* | *ApoE-/-L-sel-/-* | *ApoE-/-* | *ApoE-/-L-sel-/-* |
| **cholesterol** | 9.16 ± 1.19 | 10.48 ± 0.77 | 32.88 ± 1.53 | 30.10 ± 1.95 | 32.31 ± 2.03 | 30.78 ± 2.53 |
| **triglyceride** | 1.56 ± 0.13 | 1.73 ± 0.18 | 1.25 ± 0.19 | 1.35 ± 0.23 | 1.27 ± 0.17 | 0.94 ± 0.08 |
